# Migratory Reed Warblers Need Intact Trigeminal Nerves to Correct for a 1,000 km Eastward Displacement

**DOI:** 10.1371/journal.pone.0065847

**Published:** 2013-06-26

**Authors:** Dmitry Kishkinev, Nikita Chernetsov, Dominik Heyers, Henrik Mouritsen

**Affiliations:** 1 Arbeitsgruppe “Neurosensorik/Animal Navigation”, Institut für Biologie und Umweltwissenschaften & Research Centre for Neurosensory Sciences, University of Oldenburg, Oldenburg, Germany; 2 Biological Station Rybachy, Zoological Institute of Russian Academy of Sciences, Rybachy, Kaliningrad Region, Russia; University of Auckland, New Zealand

## Abstract

Several studies have shown that experienced night-migratory songbirds can determine their position, but it has remained a mystery which cues and sensory mechanisms they use, in particular, those used to determine longitude (east–west position). One potential solution would be to use a magnetic map or signpost mechanism like the one documented in sea turtles. Night-migratory songbirds have a magnetic compass in their eyes and a second magnetic sense with unknown biological function involving the ophthalmic branch of the trigeminal nerve (V1). Could V1 be involved in determining east–west position? We displaced 57 Eurasian reed warblers (*Acrocephalus scirpaceus*) with or without sectioned V1. Sham operated birds corrected their orientation towards the breeding area after displacement like the untreated controls did. In contrast, V1-sectioned birds did not correct for the displacement. They oriented in the same direction after the displacement as they had done at the capture site. Thus, an intact ophthalmic branch of the trigeminal nerve is necessary for detecting the 1,000 km eastward displacement in this night-migratory songbird. Our results suggest that V1 carries map-related information used in a large-scale map or signpost sense that the reed warblers needed to determine their approximate geographical position and/or an east–west coordinate.

## Introduction

Several displacement experiments to unfamiliar locations [1]–[5] and high site fidelity rates ([6], [7]; see [8] for a review) strongly suggest that night-migratory songbirds having completed at least one migration are able to perform true navigation, i.e. they must have both a compass and a map [9]–[13]. The compasses used by night-migratory birds are relatively well understood [14]–[19], whereas the map mechanisms remain enigmatic and hotly debated. It is easy to explain how a bird would be able to detect its north-south position from celestial cues by using, for example, the height of the Sun disk at the zenith or the center of rotation of the stellar sky. In contrast, no global cue(s), which birds are known to be able to sense, change regularly along the East-West axis [20].

**Figure 1:**
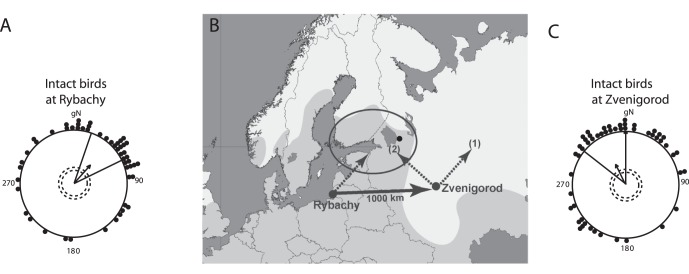
Results of our previous displacement study with intact Eurasian reed warblers (re-drawn after [4]). (*A*) Orientation of birds at the capture site (Rybachy). (*C*) Orientation of the same birds after the 1,000 km eastward translocation at the displacement site (Zvenigorod). On *A* and *C*, pooled data for 2004, 2005 and 2007 are shown. Each dot at the circular diagram periphery indicates the mean orientation of one individual bird. The arrows show group mean directions and vector lengths. The dashed circles indicate the length of the group mean vector needed for significance (5% and 1% level for inner and outer dashed circle, correspondingly) according to the Rayleigh test [21]. The lines flanking group mean vectors give 95% confidence intervals. gN – geographic North. On (*B*), a map of the displacement is shown. The shaded light gray zone represents the breeding range of the Eurasian reed warbler. Visual observations by local ornithologists (e.g. [22]) confirm that there are no regular breeding populations of Eurasian reed warblers further east than indicated on the map. The black filled circle represents the single known recovery of a reed warbler ringed in Rybachy and re-captured as a breeding bird [23], [24]. The dashed line vector from the capture site at Rybachy shows the mean migratory direction of the given species according to our previous study ([4], α = 42°). The solid line circle represents a proposed area where transit Eurasian reed warblers are heading to based on our previous study [4] combined with ringing recoveries (the goal). The solid line vector from Rybachy to Zvenigorod shows the direction and distance of the displacement. The two dashed line vectors from Zvenigorod represent our expectations for V1-sectioned and sham-sectioned birds, respectively: (1): no compensation, (2): compensation towards the eastern part of the breeding range.

Nevertheless, recent East-West displacement experiments [3], [4] have shown that several species of night-migratory birds can correct their orientation towards their breeding or wintering areas and thus can compensate for longitudinal displacements. For example, non-treated Eurasian reed warblers oriented towards the Northeast in Rybachy, Eastern Baltic (Figure 1*A*, orientation data from [4]). Following a 1,000 km eastward displacement to Zvenigorod, Moscow region, the same birds oriented towards the North-Northwest (Figure 1*C*, orientation data from [4]), which indicates a clear corrective orientation towards the Northeastern part of their breeding range (Figure 1*B*). This change in orientation, following a displacement due east, means that Eurasian reed warblers must have detected the displacement. Since the birds were displaced to a completely unknown location and were tested in orientation funnels, the used map sense cannot have been based on local cues like landmarks, but must have been based on at least regionally, if not globally, available cues. What cues do Eurasian reed warblers use for such a map sense?

Recently, we tested whether a time zone or jet lag effect may serve as a map sense, but an exposure to the time difference alone did not lead to compensatory re-orientation [20]. The stars are also unlikely to provide useful east-west positional information to night-migratory songbirds [25]. Therefore, the question about the cue(s) that night-migratory songbirds use to determine their east-west position has remained elusive.

Another potential way to obtain information about position would be to use magnetic field parameters. It is currently widely accepted that birds possess at least two (maybe even three [26], [27], for a review see [28]) magnetosensory systems: (i) a visually based magnetosensor assumingly based on radical-pair-forming molecules in the retina [29]–[33] and (ii) a magnetoreceptor innervated by the ophthalmic branch of the trigeminal nerve (V1) assumingly iron-oxide-based and located in the upper beak [34]–[39] but see [40], [41], and see [28] concerning the problem of repeatability of the references [34], [35].

Behavioral studies in European robins [33] strongly suggest that a forebrain region named “Cluster N” [31], which receives input from the eyes via the thalamofugal visual pathway [32], is involved in processing magnetic compass information. Birds with inactivated Cluster N cannot use their magnetic compass, but their sun and star compasses remain functional [33]. In contrast, anesthetizing [42] or bilaterally dissecting [33] of V1 nerves did not affect the magnetic compass orientation abilities in bobolinks [42] and in European robins [33]. Thus, V1 is neither necessary nor sufficient for transmitting magnetic compass information.

Electrophysiological recordings from V1 and the trigeminal ganglion of the bobolink (*Dolichonyx oryzivorus*) by Beason and Semm claimed that V1 transmits magnetic information [34], [35], but the relevance of these studies is questionable, since, despite several serious attempts, nobody has managed to replicate them [28]. More interestingly, in two recent independent studies, migratory European robins [39] and non-migratory homing pigeons [26] were exposed to changing magnetic stimuli, and neuronal activity in the V1-recipient regions in the hindbrain was quantified using ZENK [39] or c-fos [26] expression. The results suggest that neurons in the trigeminal nuclei are most probably activated by magnetic stimuli. Thus, V1 seems to transmit magnetic information into the brain in different avian species, but the biological function of this information remains unknown [39]. It has often been assumed that this information is perceived by a putative magnetic map organ associated with the trigeminal nerve [36]–[39] even though the receptors remain to be identified with certainty [40], [41].

The biological function of V1-transmitted information for navigational performance has never been directly shown in an experiment. Could a magnetic sense based on the detection of differences in geomagnetic field parameters and transmitting information through the ophthalmic branch of the trigeminal nerve allow birds to detect their eastward displacement? If this would be the case, birds with bilaterally sectioned V1 should not be able to compensate for an east-west displacement. Therefore, the aim of the present study was to test whether bilaterally intact V1 is necessary to enable Eurasian reed warblers to adjust their orientation towards the breeding area after a 1,000 km eastward displacement.

To test this, we captured migrating Eurasian reed warblers on spring migration at Rybachy in the Eastern Baltic, tested their control orientation in Emlen funnels [43], bilaterally cut the ophthalmic branch of the trigeminal nerve or did equivalent sham surgery, displaced all birds to Zvenigorod, which lies east of any known breeding location for Eurasian reed warblers above 55° latitude. In Zvenigorod, we tested the birds again in Emlen funnels and compared their orientation after the displacement with the same birds’ orientation prior to the displacement and with the orientation of the non-operated birds displaced previously [4]. The possibility to displace Eurasian reed warblers from our capture site at the Eastern Baltic coast (Rybachy, Kaliningrad region [55°09′N, 20°52′E], Figures 1*B* *,* 2*D*) due east to Zvenigorod (Moscow region [55°42′N, 36°45′E], Figures 1*B* *,* 2*D*) is close to ideal for this study, because there is significant variation in magnetic parameters between these two sites (Figure 3). Both magnetic intensity and magnetic inclination, which sea turtles are known to use as part of their magnetic map [44]–[46], are increasing from west to east in this region (Figures 3*A, B*).

**Figure 2:**
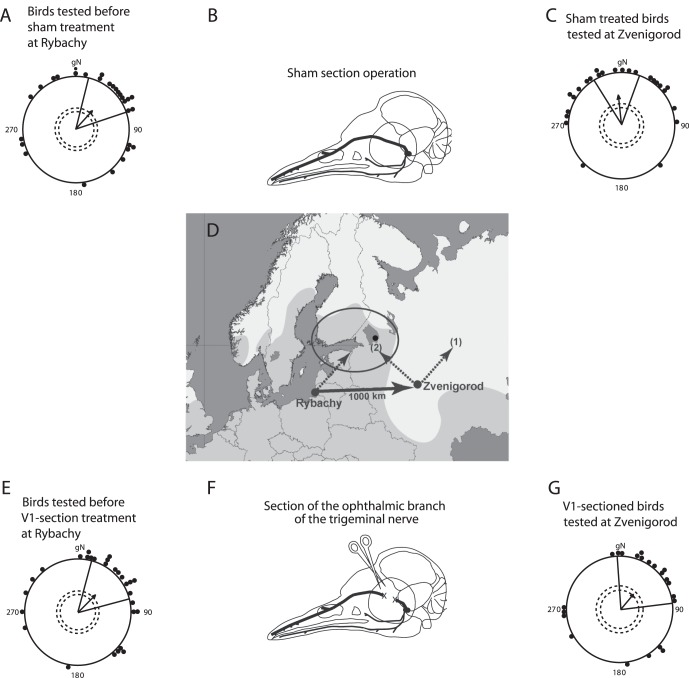
Orientation of birds at the capture and displacement site. (*A, C*) Results for sham-sectioned birds, before sham-surgery at the capture site (*A*) and after sham-surgery and translocation to the displacement site (*C*). (*E, G*) Results for V1-sectioned birds before V1-sectioning at the capture site (*E*) and after V1-sectioning surgery and translocation to the displacement site (*G*). For description of the circular diagrams and the map (*D*), see Figure 1. The schemes of real V1-section (*F*) and sham section (*B*) show the approximate locations of the three branches of the trigeminal nerve. The ophthalmic branch (V1) is shown in bold. The crosses on *F* indicate the approximate locations at which the nerve was sectioned and a piece of the nerve was removed. For details about the surgeries, see Methods.

**Figure 3:**
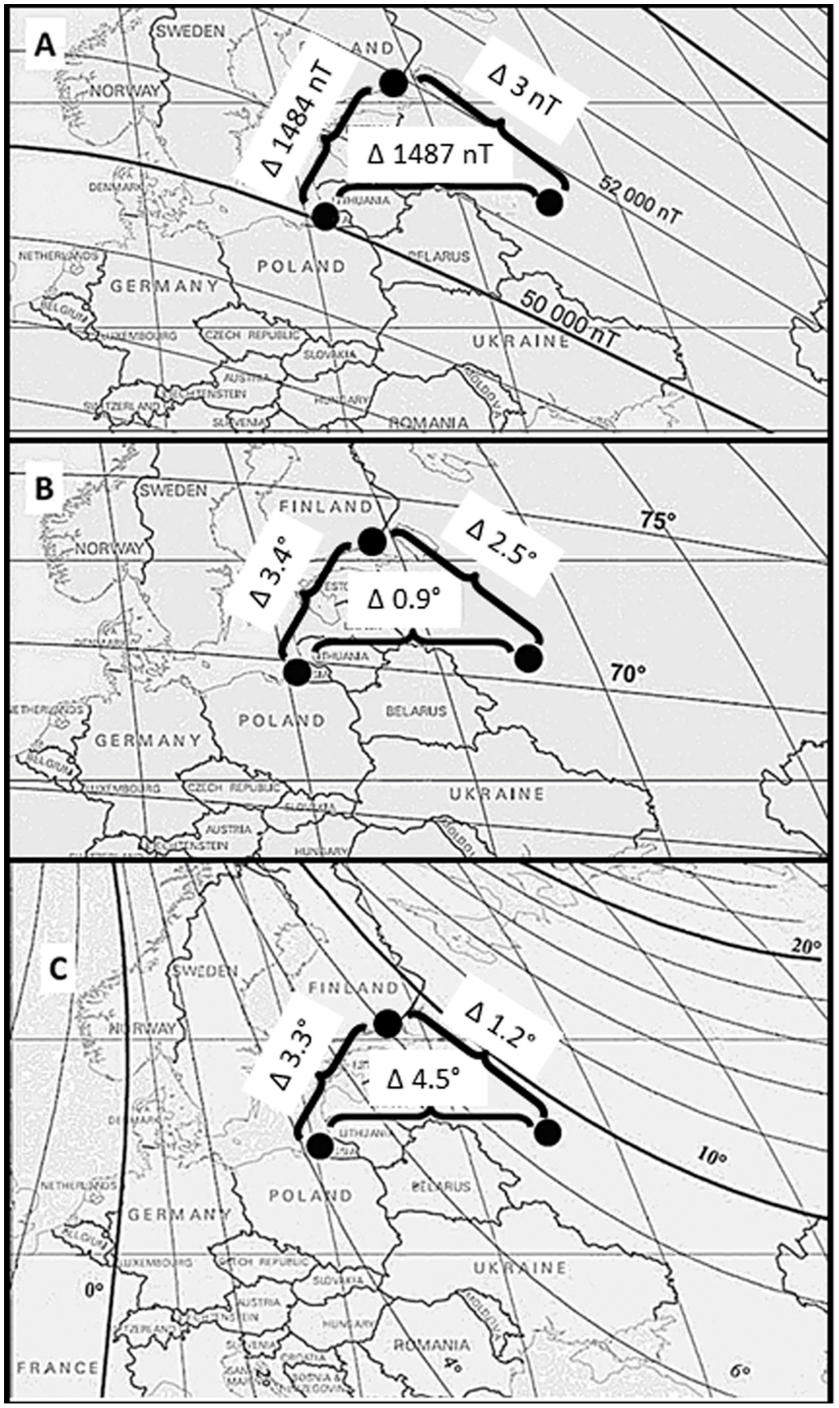
Differences of the geomagnetic field parameters between capture site, displacement site and a putative goal area. (*A*) Difference in total intensity; (*B*) Difference in inclination; (*C*) Difference in declination. The measured geomagnetic field parameters at Rybachy (the left dot) were the following: total intensity 50,688 nT, inclination 70.3°, declination 5.6°. The measured geomagnetic field parameters at Zvenigorod (the right dot) were the following: total intensity 52,175 nT, inclination 71.2°, declination 10.1°. As a goal site (the upper dot), the centroid of “the goal” shown on Figure 1 (60° 30′N, 27° 51′E) was taken. Computation of the Earth’s magnetic field parameters for the goal site was done with the calculator of IGRF Model 11 in the website of the National Geophysical Data Center (http://www.ngdc.noaa.gov/geomag/geomag.shtml). The calculated geomagnetic field parameters at the goal site were the following: total intensity 52,172 nT, inclination 73.7°, declination 8.9°. The charts with the isolines of the geomagnetic field parameters are taken from http://pubs.usgs.gov/sim/2007/2964 with modifications.

## Results

A total of 57 Eurasian reed warblers captured during spring migrations 2010 and 2011 in Rybachy and tested in Emlen funnels under clear natural sky were oriented in the expected [4], [23], [24] Northeastern direction (*α* = 44°, *r* = 0.47, *n* = 57, *P*<0.001, 95% confidence interval of a mean group direction [CI_mean] = 23°–65°, Figures 2*A* *+*2*E*). After the orientation tests at the capture site, and prior to the displacement, we split the birds into two groups with very similar circular distributions. In one group, the ophthalmic branch of the trigeminal nerve was cut bilaterally (V1 sectioned birds, Figure 2*E*, orientation of these individuals in Rybachy prior to the operation: *α* = 45°, *r* = 0.47, *n* = 28, *P* = 0.002, CI_mean = 15°–75°). In another group, the same surgical treatment was undertaken but the ophthalmic branch of the trigeminal nerve was left bilaterally intact (sham-sectioned birds, Figure 2*A*, orientation of these individuals in Rybachy prior to the operation: *α* = 43°, *r* = 0.48, *n* = 29, *P* = 0.001, CI_mean = 14°–72°). The total of 109 individual intact birds we tested in Rybachy prior to the displacements during 2004, 2005, 2007 (data for these years were published in [4]), 2010, and 2011 (data presented here) shows that reed warblers in Rybachy very consistently orient towards the NE in all years (Figures 1*A* *+*2*A+*2*E*: *α* = 43°, *r* = 0.47, *n* = 109, *P*<0.001, CI_mean = 27°–58°).

After the surgeries, both groups of birds were displaced 1,000 km eastwards to Zvenigorod (Figures 1*B*, 2*D*) and tested with Emlen funnels under the natural starry sky. During their time in Zvenigorod, the birds had access to the natural day length (light-dark) information, natural celestial cues and the local odors. The experimenters who did all the orientation tests and analyzed the orientation data did not know which operation (real or sham) each bird had experienced until all behavioral tests and data analyses were finished. Thus, the experiment was done double blindly.

After being displaced to Zvenigorod, the sham-sectioned birds shifted their orientation by 49° counterclockwise compared to the same birds’ orientation in Rybachy, and they oriented slightly west of North (Figure 2*C*, *α* = 354°, *r* = 0.56, *n* = 25, *P*<0.001, CI_mean = 328° –20°). This direction is significantly different from the direction shown by all intact reed warblers tested in Rybachy prior to the displacements (Figure 2*C* compared to Figures 1*A* *+*2*A+*2*E*, MWW test: *W* = 7.80, *P* = 0.02), and is not significantly different from the NNW-orientation shown by the displaced non-operated birds from the previous study ([4], Figure 2*C* compared to Figure 1*C*, MWW test: *W* = 0.36, *P* = 0.84). Furthermore, the 95% CI for the group mean direction of the sham operated birds after displacement (328° –20°) does not include either the group mean direction of V1-sectioned birds after displacement (40°, Figure 2*G*) or the group mean orientation of the sham operated birds prior to displacement (43°, Figure 2*A*). Thus, the sham-sectioned birds at the displacement site behaved like the intact birds by correcting for the displacement towards their breeding area.

In contrast, the V1-sectioned birds displaced to Zvenigorod did not correct for the displacement. They oriented towards the Northeast in Zvenigorod (Figure 2*G*, *α* = 40°, *r* = 0.38, *n* = 22, *P* = 0.04, CI_mean = 356° –83°). This orientation is almost identical to the orientation of all intact birds tested in Rybachy prior to displacement (Figure 2*G* compared to Figures 1*A*+2*A*+2*E*, Mardia-Watson-Wheeler [MWW] test: *W = *0.62, *P = *0.73). Furthermore, the orientation of the V1-sectioned birds was significantly different from the NNW-orientation shown by the displaced non-operated birds (Figure 2*G* compared to Figure 1*C*, MWW test: *W* = 6.91, *P* = 0.03) and by the displaced sham-sectioned birds (Figure 2*G* compared to Figure 2*C*, Watson-Williams F-test: *F* = 3.97, *P* = 0.05). Thus, cutting of V1 results in significantly different orientation compared to both untreated and sham-operated controls.

The numerical results are summarized in Table 1 (see also “Statistics” in “Methods” below for details).

**Table 1:** Comparison of experimental groups.

Group (Site)	Circular statistic				Comparison of groups			
	*Α (95% CI)*	*r*	*n*	*P*	*Intact (Ryb)*	*Intact (Zven)*	*V1-sect (Zven)*	*Sham sect (Zven)*
***Intact (Ryb)***	43° (27°–58°)	0.47	109	<0.001	–	W = 19.71,P<0.001	W = 0.62,P = 0.73	W = 7.80,P = 0.02
***Intact (Zven)***	334° (308°–360°)	0.41	52	<0.001	W = 19.71,P<0.001	–	W = 6.91,P = 0.03	W = 0.36,P = 0.84
***V1-sectioned (Zven)***	40° (356°–83°)	0.38	22	0.04	W = 0.62,P = 0.73	W = 6.91,P = 0.03	–	F = 3.97P = 0.05
***Sham-sectioned (Zven)***	354° (328°–20°)	0.56	25	<0.001	W = 7.80,P = 0.02	W = 0.36,P = 0.84	F = 3.97P = 0.05	–

Intact birds tested in Rybachy (Ryb) include both individuals tested in 2004, 2005 and 2007 [4] and those tested in 2010 and 2011 before surgical treatments (Figures 1*A* *+*2*A*). Intact birds tested in Zvenigorod (Zven) include individuals tested in 2004, 2005 and 2007 ([4], Figure 1*B*). For V1-sectioned (Zvenigorod) and Sham sectioned (Zvenigorod) birds see Figure 2*G and 2C*, correspondingly.

## Discussion

Our results strongly suggest that V1-sectioned birds were unable to detect the 1,000 km eastward displacement from Rybachy to Zvenigorod, whereas non-operated birds and sham-sectioned birds seem to have compensated for the displacement by correcting their orientation towards the breeding area. Thus, migrating Eurasian reed warblers seem to need an intact ophthalmic branch of the trigeminal nerve to determine this 1,000 km eastward displacement. As a consequence, it is highly likely that some sort of map information input critical to determining some aspect of position in these birds is transmitted through V1, and that the upper beak region and/or other regions innervated by V1 contain map-related sensors. The sensory nature of this map-related information and the identity of the putative sensors perceiving it remain unknown.

Which organs and receptors can V1 innervate? V1 provides somatosensory information from the dorso-frontal part of the bird’s head including the upper beak, palate, nasal cavity and the orbital region (e.g. [47]–[49]). Somatosensory receptors perceive very local mechanical, thermal, chemical and noxious information [49], and it is hard to imagine how these local mechanical, thermal or pain factors can be used as navigational cues. In addition to the somatosensory receptors, V1 most likely transmits information from two other types of receptors: magnetoreceptors [37], [39] and/or chemical/olfactory receptors located in the olfactory epithelium [50]–[54], which are more likely to provide map-relevant information. Therefore, these two possibilities are now discussed in more detail.

Based on the current literature, we suggest that the most likely kind of map information that could be transmitted via the ophthalmic branch of the trigeminal nerve is some sort of magnetic information. There is quite a bit of evidence suggesting that the ophthalmic branch of the trigeminal nerve may carry magnetic information [37], [39], which can be relevant for detection of geographical position. Most importantly, magnetic stimulation is known to activate the hindbrain regions receiving primary input through V1 in migratory European robins [39], and sectioning of V1 in homing pigeons prevents them from detecting a strong magnetic anomaly in a conditioning paradigm [37]. Our findings are also, in principle, consistent with earlier electrophysiological claims [34], [35]. However, the relevance of these studies is questionable, since, despite several serious attempts, nobody has been able to replicate them [28]. Furthermore, several reports show that brief magnetic pulses strong enough to re-magnetize magnetite or other magnetized particles without rotating them, when applied to a bird’s head, can lead to deflected orientation in adult migratory birds, both when tested in Emlen funnels [55]–[57] and when performing natural migratory flights [58]. Juvenile birds before their migration seem not to be affected by such magnetic pulse treatment, probably because of their lack of navigational experience [59], [60]. It has often been hypothesized that this deflection effect in experienced avian migrants can be due to a distorted function of a map organ containing magnetized particles [36]–[39] but see [40], [41]. However, it is still uncertain how a strong magnetic pulse affects a bird’s magnetic and other sensory receptors. Interestingly, in adult bobolinks – New World trans-equatorial avian migrants – the deflection effect of the strong magnetic pulse disappears, and birds show their normal (non-deflected) migratory direction when the ophthalmic branch of the trigeminal nerve was bilaterally anaesthetized [42]. This study by Beason and Semm [42] is in line with our present data because in both cases migratory birds show stereotyped compass orientation behaviour if they lack the information transmitted via V1. The main differences between our study and that of Beason and Semm [42] are that our birds showed a predictable and adaptive shift in orientation after a long-distance physical displacement, whereas the bobolinks showed an unexplained shift in orientation after being exposed to a strong magnetic pulse, and a potentially non-specific anaesthetics treatment was used in [42] compared to actual nerve cuts in the present experiment. In both cases, however, one could speculate that birds returned to simple “back-up” compass orientation [12], [13], [61] when V1-transmitted map-relevant information is no longer available.

At first glance, our results may seem to contradict a study by Holland et al. [62]: magnetically pulsed gray catbirds (*Dumetella carolinensis*) compensated for a displacement from Illinois to New Jersey as well as intact control birds did. In contrast, displaced anosmic catbirds seemed unable to compensate for displacement and reverted to directional orientation similar to that shown by juvenile catbirds making their first migration.

We are not surprised that olfactory cues may provide very important map information under some circumstances, and we would like to stress that the complete map/positioning determining system in free-flying migrants is almost certainly multifactorial. It most likely receives inputs from all potentially useful senses including at least olfaction, vision and magnetoperception. The relative importance of the different inputs/cues is likely to vary depending on the navigational scale and the concrete ecological context. However, in the reed warbler system, we studied here, some sort of cue(s) sensed via the ophthalmic branch of the trigeminal nerve seem(s) to play a major role, and since our displaced reed warblers were exposed to the natural odors of Zvenigorod and their olfactory nerves were left intact, odor cues are unlikely to have been the major contributing cue. Nevertheless, it cannot be completely ruled out that some odor cues could be transmitted via the ophthalmic branch of the trigeminal nerve. The reason for this is that there are anatomical and behavioral data suggesting that different vertebrates have trigeminal fibers innervating the olfactory epithelium [50]–[52]. Furthermore, iron-positive cells located in the olfactory epithelium have been found in rainbow trouts (*Oncorhynchus mykiss,* [53]), in which electrophysiological responses in the superficial ophthalmic ramus of the trigeminal nerve (ros V) have been recorded after magnetic field stimulation. In birds, trigeminal innervation of the nasal cavity and the putative involvement of the trigeminal nerve in perception of odors have been scarcely studied. In the European starling (*Sturnus vulgaris*), it has been suggested that trigeminal nerve terminals encode for chemical irritation and pain, and are involved in the avoidance behavior [54]. Thus, because of general anatomical similarity of cranial nerves in vertebrates, it is possible that birds have trigeminal innervation of the olfactory epithelium and therefore could potentially perceive olfactory stimuli via the trigeminal nerve. The other way around, it is also possible that the olfactory epithelium could harbor magnetic sensors communicating to the brain via V1.

Further along these lines, it might appear contradictory that the map of Eurasian reed warblers seems to depend strongly on intact V1, whereas homing pigeons are able to navigate after V1-sectioning [63]–[65] or the upper beak’s skin anaesthesia [66]. This may be due to species-specific differences, but more likely might hint at the important effect of scale. The pigeons used in the studies by Gagliardo et al. [63]–[65] were released ca. 50 km from their home lofts, whereas our Eurasian reed warblers were displaced ca. 1,000 km from their capture site. Why could this difference in displacement distances be important?

The geomagnetic field changes ca. 30,000 nanoTesla (nT) from the magnetic poles to the magnetic equator. The distance from the North Pole to the equator is ca. 10,000 km. Consequently, the average change in magnetic field strength is ca. 3 nT/km on the North-South axis (there are, of course, variability in some areas, but the general trend is very consistent). Magnetic intensity usually changes much less on the East-West axis. On top of this basic field, there are somewhat regular but still to a large degree stochastic, unpredictable variations in geomagnetic field strength within a day and between days of typically ±30–100 nT. We therefore speculate that a magnetic map or signpost sense (simple threshold values rather than a detailed magnetic map might be sufficient to trigger adaptive changes in behavior) of birds, if exists, may work only at relatively large distances where the expected differences in the magnetic field parameters are consistently larger than the daily noise in these same parameters, whereas other cues such as odors [63]–[65], [67]–[69] and landmarks [70], [71] may likely be more significant map parameters at the shorter distances typically used in pigeon homing experiments. Odor and landmark cues are not likely to be easier to detect on the north-south axis than on the east-west axis, and it is therefore not surprising that there is no evidence that pigeons have more difficulties with east-west displacements than with north-south displacements (see [72] for a review).

It has previously been suggested that geomagnetic field parameters or their combination (e.g., inclination and total intensity) may serve as coordinates in homing pigeons [73], [74], migratory birds [75], [76] and sea turtles [44]–[46]. If our Eurasian reed warblers include magnetic field parameters in their position determination system, which parameters of the Earth’s magnetic field could they use?

No geomagnetic field parameters are consistently distributed across the surface of the Earth, and in many regions their isoclines do not make a proper grid [77], [78]. Thus, geomagnetic field parameters cannot provide the basis for a globally functional map sense. However, the maps of animals do not need to work on the global scale. They need only to work in the ranges typically navigated by any given animal species. For example, in Western Europe, the large scale isoclines of both magnetic intensity and magnetic inclination run almost parallel to geographic latitudes (Figures 3*A*+*B*, left sides). It is therefore very difficult, if not impossible, to detect east-west displacement in Western Europe based on these magnetic parameters. If, however, magnetic declination is considered (Figures 3*A*+*C* and Figures 3*B*+*C*) a potentially useful grid may appear. To measure the declination, birds theoretically would have to combine magnetic compass information with information from celestial reference(s) that would enable the birds to detect geographic North (e.g. the location of the North Star [15] and/or averaging of positions of the setting and rising sun [79]). When a bird moves (or is displaced) further into Eastern Europe or Siberia, magnetic intensity and inclination start forming a potentially useful grid (Figures 3*A*+*B*, right sides). In the specific case of our experiment, all parameters of the Earth’s magnetic field were different between the capture and displacement site with a general tendency for values to increase from west to east: total magnetic intensity is increasing by ∼1500 nT (Figure 3*A*), magnetic inclination is increasing by ∼1° (Figure 3*B*) and magnetic declination is increasing by 4.5° (Figure 3*C*). Can birds detect such small differences in these parameters? We do not know for sure at the moment. However, it has been suggested that birds may be able to detect very small changes in intensity [73], [80] and inclination [81].

In conclusion, the ability of Eurasian reed warblers, which are typical long-distance night-migratory songbirds, to correct for an eastward displacement, requires intact ophthalmic branch of the trigeminal nerve. This result suggests that some kind of map information is transmitted via the ophthalmic branch of trigeminal nerve into the brain. The sensory nature of this information is not demonstrated by the present results. However, when we consider the previous literature [1]–[81] including the fact that magnetically activated neurons have been found in the trigeminal recipient hindbrain regions [39], the ophthalmic branch of the trigeminal nerve could very well be processing magnetic information relevant for a magnetic map or signpost sense.

## Methods

### Ethics Statement

The experiments were conducted in accordance with the national animal welfare legislation of Russia. We had no federal permits because this was a regional issue. The permits No. 2010-03 and 2011-05 were issued by the responsible regional agency of Kaliningrad region, the Regional Agency for Protection, Reproduction and Use of Animal World and Forests. To alleviate suffering due to surgery, all the treatments were done under general anesthesia (see Section “Surgical treatments” below for details).

### Experimental Birds and Sites

We captured 57 Eurasian reed warblers (*Acrocephalus scirpaceus*) during their spring migration in late May – early June 2010–2011 at Rybachy (55°09′N, 20°52′E) on the Courish Spit on the southeastern Baltic coast. According to ringing recoveries, Eurasian reed warblers that migrate through Rybachy in spring breed to the northeast of the study site in the Baltic countries, Finland, and north-western Russia [23], [24]. In order to increase the probability of testing only transient migrating birds breeding further north of the capture site, we only selected individuals with subcutaneous fat scores ≥2 [82] because it was shown that such fat Eurasian reed warblers are most probably not local birds, who normally arrive with low fat deposits [83]. At the capture site on the Courish Spit, the birds were kept indoors in individual cages (60×20×20 cm or 40×20×20 cm) under the local photoperiod exposed to natural full spectrum light coming in through windows.

At the displacement site in Zvenigorod, the birds were kept in individual cages in a room with windows under the natural local photoperiod. Thus, natural full spectrum light was available to the birds. Holding conditions (cage size, light, food, water) at the capture site and at the displacement sites were very similar. Before orientation tests, both at the capture site and at the displacement site, we let the birds observe the sky during sunset. To do this, we placed them outdoors in their cages 1 h before sunset and put them back into the room 1 h after the sunset. This was done for the facilitation of compass calibration [16], [79] but see [84]. The handling of the birds in both Rybachy and Zvenigorod allowed them to smell any local odors. Orientation tests in Zvenigorod and data analysis were done in exactly the same manner as in Rybachy. The birds were provided with food (mealworms) and water *ad libitum*.

### Orientation Tests

In total, 388 orientation tests were performed with Emlen funnels [43] outdoors under clear starry skies. All tests were performed when at least 50% of the starry sky was visible; in most tests, it was 95% - 100% clear. We did 1–3 tests per bird at Rybachy for control directions of intact birds and 3–5 tests per bird at Zvenigorod for directions of surgically treated and displaced birds. Because not every night had suitable weather conditions we had 0–2 day pauses between tests. Each test lasted for 40 min and started at the beginning of astronomical twilight. We used modified Emlen funnels made of aluminium (top diameter 300 mm, bottom diameter 100 mm, slope 45° with the top opening covered by netting). The directionality of the birds’ activity was recorded as scratches left by their claws as they hopped in the funnels on a print film covered with a dried mixture of whiting and glue. Two researchers (DK and NC) independently determined each bird’s mean direction from the distribution of the scratches. In most cases, the mean direction could be very precisely identified using the simple visual estimation method ([85]; routinely used in previous studies, e.g. [17]–[19], [33]). If a pattern of scratches was not clear, both persons independently counted scratches in each of 36 10° sectors (in active birds, there are so many scratches that an exact number of scratches cannot be counted, but must be estimated) and used circular statistics software to assess the directionality based on the numbers of scratches. As a result, both persons obtained their own mean directions and compared them. The mean of the two observer’s determined directions was recorded as the orientation result. If both observers considered the scratches to be randomly distributed or if the two mean directions deviated by more than 30°, the bird was considered to be not oriented in the given test. The result of a given test was included only if at least 40 scratches were visible on the print film and if a unidirectional mean direction was statistically significant (Rayleigh test, [21]). Inactive (fewer than 40 scratches) tests (29 (7.4%) of all testes conducted) and disoriented (mean direction is not significantly oriented by the Rayleight test, [21]) tests (130 (33.5%) of all testes conducted) were excluded from analysis.

We did not do orientation tests at the capture site after the surgeries because of shortage of time. We considered it most important to make sure that there were no differences in initial orientation between the non-manipulated birds from our previous study [4], the sham-operated group, and the V1-sectioned group. In order to be sure about this, we had to test the birds un-manipulated and split them in two equally orienting groups before the operations. Eurasian reed warblers are late migrants and normally trapped between May 20^th^ and 25^th^, and their migratory restlessness in captivity was waning in early June and rapidly ceased after June 10^th^–12^th^. Therefore, we had no time available for further pre-displacement tests. However, pre-displacement tests with the operated birds are not crucial to our design because we also displaced sham-operated birds and because we know that dissection and/or anesthesia of V1 nerves in European robins and bobolinks [33], [42] do not affect their ability to orient in their normal migratory compass direction. The sham-operated birds tested after the displacement served as controls for any effects of the surgery procedure itself. Since the sham-operated birds corrected for the displacement as the displaced, intact, non-manipulated birds did (Table 1), the seen effect is due to the cutting of the nerve, not the operational procedure itself.

### Surgical Treatments

After the orientation tests at the capture site at Rybachy, all birds that showed at least one significant control direction were divided into two experimental groups with the same number of individuals. To avoid any directional biases in our experimental groups, we ranked all the birds’ azimuths on a circular diagram from 0° to 359° clockwise and assigned each bird with an odd rank to one group and each bird with an even rank to another one. One group – *real V1-sectioned birds* – underwent bilateral section and removal of a relatively large part (approx. 3–5 mm) of the ophthalmic branch of the trigeminal nerve (V1, Figure 2*F*). Removal of a piece of V1 is a very reliable method tested in previous studies with identical surgical treatment where absence of re-growth was confirmed after a few weeks (e.g. [33], [39]). To re-confirm that the nerves did not re-grow during the experiment, 5 birds in 2010 were sacrificed after the experiment, their heads were examined, and the lack of re-growth was confirmed in all cases. Another group – *sham-sectioned birds* – underwent exactly the same surgical procedure except that V1s were left intact (Figure 2*B*). The sham group was included to make sure that birds were visually undistinguishable as to operation condition and to test for any effect on the birds’ behavior by the surgery itself. Birds were anaesthetized using intramuscular injection of Medetomidine (Domitor©, 0.1%; 0.1 ml/kg body weight)+Ketamine (10%; 0.1 ml/kg body weight) and immobilized using a custom built holder. Access to V1 was gained through a small incision along the dorsal rim of the orbit and gentle retraction of the eyeball and oculomotor muscles. To minimize the duration of anaesthesia, the effect of Medetomidine was antagonized using Atipamezol (Antisedan©, 0.5%; 0.1 ml/kg body weight) at the end of the surgery. Only the person who did all the surgical treatments (DH), but not the experimenters who did all orientation tests (DK and NC), was aware which bird belonged to which group. Thus, orientation tests and analysis of Emlen funnel data were performed double-blindly. Each bird was given at least 5 days to recover from the surgery before participating in any orientation tests at the displacement site.

### Displacement of the Birds

After the surgical treatments, the birds were displaced from the capture site almost due east to Zvenigorod Biological Station of the Moscow State University, 40 km west of Moscow (55°42′N, 36°45′E). The displacement distance was 1,004 km (Figures 1*B*, 2*D*). The translocation was done by air (a direct flight from Kaliningrad to Moscow Airport Sheremetyevo, approx. 2 h), train (from Airport Sheremetyevo to Moscow Belorussky Railway Station, 45 min, and then to Zvenigorod Railway Station, approx. 2 h) and car (from Biological Station Rybachy to Kaliningrad Airport Khrabrovo, approx. 1 h, and from Zvenigorod Railway Station to Zvenigorod Biological Station, approx. 20 min). The whole translocation procedure took around 12–14 h. All birds in both years survived the displacement. Eurasian reed warblers that migrate through Rybachy in spring do not breed further east than the southeastern coast of Lake Ladoga, which is 33°–34°E [22]. Therefore, after the displacement, the birds were almost certainly southeast of their intended breeding area. If the birds would compensate their orientation towards breeding area after the displacement, they should therefore show northwesterly orientation, as did non-operated birds displaced previously [4].

### Statistics

Differences in mean direction between experimental groups were analyzed using the parametric Watson-Williams F-test in the cases where the assumptions underlying this test were fulfilled (this is automatically tested by the newest version of the circular statistics program “Oriana” (version 4.01)). When the concentrations of the samples to be compared were too low to use the Watson-Williams F-test, the nonparametric Mardia-Watson-Wheeler test was used [21].

## References

[1] Perdeck AC (1958) Two types of orientation in migrating starlings, *Sturnus vulgaris* L., and chaffinches, *Fringilla coelebs* L., as revealed by displacement experiments. Ardea 46: 1–37.

[2] Mewaldt R (1964) California sparrows return from displacement to Maryland. Science 146: 941–942. 17777062 10.1126/science.146.3646.941

[3] Thorup K, Bisson I-A, Bowlin MS, Holland RA, Wingfield JC, et al (2007) Evidence for a navigational map stretching across the continental U.S. in a migratory songbird. Proc Natl Acad Sci USA 104: 18115–18119. 17986618 10.1073/pnas.0704734104 PMC2084305

[4] Chernetsov N, Kishkinev D, Mouritsen H (2008) A long-distance avian migrant compensates for longitudinal displacement during spring migration. Curr Biol 18: 188–190. 18249113 10.1016/j.cub.2008.01.018

[5] Perdeck A (1983) An experiment on the orientation of juvenile starlings during spring migration: an addendum. Ardea 71: 255.

[6] Löhrl H (1959) Zur Frage des Zeitpunktes einer Prägung auf die Heimatregion beim Halsbandschnäpper (*Ficedula albicollis*). J Ornithol 100: 132–140.

[7] Sokolov LV, Bolshakov CV, Vinogradova NV, Dolnik TV, Lyuleeva DS, et al (1984) The testing of the ability for imprinting and finding the site of future nesting in young Chaffinches. Zool Zhurnal 63: 1671–1681.

[8] Sokolov LV (1997) Phylopatry of migratory birds. In: Turpaev TM, editor. Physiology and general biology reviews. Amsterdam: Harwood Academic Press. 1–58.

[9] Kramer G (1953) Wird die Sonnenhöhe bei der Heimfindeorientierung verwertet? J Ornithol 94: 201–219.

[10] Kramer G (1957) Experiments on bird orientation and their interpretation. Ibis 99: 196–227.

[11] Rabøl J (1978) One-direction orientation versus goal area navigation in migratory birds. Oikos 30: 216–223.

[12] Berthold P (1991) Spatiotemporal programmes and genetics of orientation. In: Berthold P, editor. Orientation in birds. Berlin: Birkhäuser. 86–105. 10.1007/978-3-0348-7208-9_5 1838524

[13] Mouritsen H (2003) Spatiotemporal orientation strategies of long-distance migrants. In: Berthold P, Gwinner E, Sonnenschein E, editors. Avian Migration. New York: Springer. 493–513.

[14] Wiltschko W, Wiltschko R (1972) Magnetic compass of European robins. Science 176: 62–64. 17784420 10.1126/science.176.4030.62

[15] Emlen ST (1975) The stellar-orientation system of a migratory bird. Sci Am 233: 102–111. 1145171 10.1038/scientificamerican0875-102

[16] Cochran WW, Mouritsen H, Wikelski M (2004) Migrating songbirds recalibrate their magnetic compass daily from twilight cues. Science 304: 405–408. 15087541 10.1126/science.1095844

[17] Hein CM, Zapka M, Heyers D, Kutzschbauch S, Schneider N-L, et al (2010) Night-migratory garden warblers can orient with their magnetic compass using the left, the right or both eyes. J R Soc Interface 7: S227–S233 doi:10.1098/rsif.2009.0376.focus 19889693 10.1098/rsif.2009.0376.focus PMC2844002

[18] Hein CM, Engels S, Kishkinev D, Mouritsen H (2011) Robins have a magnetic compass in both eyes. Nature 471: E11–E12 doi:10.1038/nature09875 21455128 10.1038/nature09875

[19] Engels S, Hein CM, Lefeldt N, Prior H, Mouritsen H (2012) Night-migratory songbirds possess a magnetic compass in both eyes. PLoS One 7: e43271 doi: 10.1371/journal.pone.0043271 22984416 10.1371/journal.pone.0043271 PMC3440406

[20] Kishkinev D, Chernetsov N, Mouritsen H (2010) A double clock or jetlag mechanism is unlikely to be involved in detection of east-west displacements in a long-distance avian migrant. Auk 127: 773–780.

[21] Batschelet E (1981) Circular Statistics in Biology. London: Academic Press. 371 p.

[22] Popelnyukh VV (2002) Ecology of *Acrocephalus* Warblers in the Southeastern Ladoga Area. St. Petersburg: St. Petersburg University Press.

[23] Bolshakov CV, Shapoval AP, Zelenova NP (2001) Results of bird trapping and ringing by the Biological Station “Rybachy” on the Courish Spit: Long-distance recoveries of birds ringed in 1956–1997: Part 1. Avian Ecol Behav Suppl 1: 1–126.

[24] Bolshakov CV, Shapoval AP, Zelenova NP (2002) Results of bird trapping and ringing by the Biological Station “Rybachy” on the Courish Spit: Controls of birds ringed outside the Courish Spit in 1956–1997. Part 1. Avian Ecol Behav Suppl 5: 1–106.

[25] Mouritsen H, Larsen ON (2001) Migrating songbirds tested in computer-controlled Emlen funnels use stellar cues for a time-independent compass. J Exp Biol 204: 3855–3865. 11807103 10.1242/jeb.204.22.3855

[26] Wu L-Q, Dickman D (2011) Magnetoreception in an avian brain in part mediated by inner ear lagena. Curr Biol 21: 418–423. 21353559 10.1016/j.cub.2011.01.058 PMC3062271

[27] Wu L-Q, Dickman DJ (2012) Neural correlates of a magnetic sense. Science 336: 1054–1057. 22539554 10.1126/science.1216567

[28] Mouritsen H, Hore PJ (2012) The magnetic retina: light-dependent and trigeminal magnetoreception in migratory birds. Curr Opin Neurobiol 22: 343–352. 22465538 10.1016/j.conb.2012.01.005

[29] Ritz T, Adem S, Schulten K (2000) A model for photoreceptor-based magnetoreception in birds. Biophys J 78: 707–718. 10653784 10.1016/S0006-3495(00)76629-X PMC1300674

[30] Mouritsen H, Janssen-Bienhold U, Liedvogel M, Feenders G, Stalleicken G, et al (2004) Cryptochromes and neuronal-activity markers colocalize in the retina of migratory birds during magnetic orientation. Proc Natl Acad Sci USA 101: 14294–14299. 15381765 10.1073/pnas.0405968101 PMC521149

[31] Mouritsen H, Feenders G, Liedvogel M, Wada K, Jarvis ED (2005) Night-vision brain area in migratory songbirds. Proc Natl Acad Sci USA 102: 8339–8344. 15928090 10.1073/pnas.0409575102 PMC1149410

[32] Heyers D, Manns M, Luksch H, Güntürkün O, Mouritsen H (2007) A visual pathway links brain structures active during magnetic compass orientation in migratory birds. PloS One 2: e937. 17895978 10.1371/journal.pone.0000937 PMC1976598

[33] Zapka M, Heyers D, Hein CM, Engels S, Schneider N-L, et al (2009) Visual but not trigeminal mediation of magnetic compass information in a migratory bird. Nature 461: 1274–1277. 19865170 10.1038/nature08528

[34] Beason RC, Semm P (1987) Magnetic responses of the trigeminal nerve system of the bobolink, *Dolichonyx oryzivorus* (Aves: *Icteridae*). Neurosci Lett 80: 229–234. 3683981 10.1016/0304-3940(87)90659-8

[35] Semm P, Beason RC (1990) Responses to small magnetic variations by the trigeminal system of the Bobolink. Brain Res Bull 25: 735–740. 2289162 10.1016/0361-9230(90)90051-z

[36] Fleissner G, Holtkamp-Rötzler E, Hanzlik M, Winklhofer M, Fleissner G, et al (2003) Ultrastructural analysis of a putative magnetoreceptor in the beak of homing pigeons. J Comp Neurol 458: 350–360. 12619070 10.1002/cne.10579

[37] Mora CV, Davison M, Wild JM, Walker MM (2004) Magnetoreception and its trigeminal mediation in the homing pigeon. Nature 432: 508–511. 15565156 10.1038/nature03077

[38] Falkenberg G, Fleissner G, Schuchardt K, Kuehbacher M, Thalau P, et al (2010) Avian magnetoreception: elaborate iron mineral containing dendrites in the upper beak seem to be a common feature of birds. PLoS One 5: e9231. 20169083 10.1371/journal.pone.0009231 PMC2821931

[39] Heyers D, Zapka M, Hoffmeister M, Wild JM, Mouritsen H (2010) Magnetic field changes activate the trigeminal brainstem complex in a migratory bird. Proc Natl Acad Sci USA 107: 9394–9399. 20439705 10.1073/pnas.0907068107 PMC2889125

[40] Treiber CD, Salzer MC, Riegler J, Edelman N, Sugar C, et al (2012) Clusters of iron-rich cells in the upper beak of pigeons are macrophages not magnetosensitive neurons. Nature 484: 367–370. 22495303 10.1038/nature11046

[41] Mouritsen H (2012) Sensory biology: search for the compass needles. Nature 484: 320–321. 22517155 10.1038/484320a

[42] Beason R, Semm P (1996) Does the avian ophthalmic nerve carry magnetic navigational information? J Exp Biol 199: 1241–1244. 9319100 10.1242/jeb.199.5.1241

[43] Emlen ST, Emlen JT (1966) A technique for recording migratory orientation of captive birds. Auk 83: 361–367.

[44] Lohmann KJ, Lohmann CMF (1994) Detection of magnetic inclination angle by sea-turtles: a possible mechanism for determining latitude. J Exp Biol 194: 23–32. 9317267 10.1242/jeb.194.1.23

[45] Lohmann KJ, Cain SD, Dodge SA, Lohmann CMF (2001) Regional magnetic fields as navigational markers for sea turtles. Science 294: 364–366. 11598298 10.1126/science.1064557

[46] Putman NF, Endres CS, Lohmann CMF, Lohmann KJ (2011) Longitude perception and bicoordinate magnetic maps in sea turtles. Curr Biol 21: 463–466. 21353561 10.1016/j.cub.2011.01.057

[47] Bubień-Waluszewska A (1981) The cranial nerves. In: King AS, McLelland J, editors. Form and function in birds. V. 2. New York: Academic Press. 385–438.

[48] Dubbeldam JL, Brauch CSM, Don A (1981) Studies on the somatotopy of the trigeminal system in the mallard, *Anas platyrhynos* L.: the afferents and organisation of the nucleus basalis. J Comp Neurol 196: 391–405. 7217363 10.1002/cne.901960304

[49] Gottschaldt K-M (1985) Structure and function of avian somatosensory receptors. In: King AS, McLelland J, editors. Form and function in birds. V. 3. New York: Academic Press. 375–461.

[50] Lang J (1989) Clinical anatomy of the nose, nasal cavity and paranasal sinuses. Stuttgart: Thieme Verlag. 149 p.

[51] Finger TE, Böttger B (1993) Peripheral peptidergic fibers of the trigeminal nerve in the olfactory bulb of the rat. J Comp Neurol 334: 117–124. 7691899 10.1002/cne.903340110

[52] Brand G (2006) Olfactory/trigeminal interactions in nasal chemoreception. Neurosci Biobehav Rev 30: 908–917. 16545453 10.1016/j.neubiorev.2006.01.002

[53] Walker MM, Diebel CE, Haugh CV, Pankhurst PM, Montgomery JC, et al (1997) Structure and function of the vertebrate magnetic sense. Nature 390: 371–376. 20358649 10.1038/37057

[54] Clark L (1996) Trigeminal repellents do not promote odor avoidance in European starlings. Wilson Bull 108: 36–52.

[55] Wiltschko W, Munro U, Beason RC, Ford H, Wiltschko R (1994) A magnetic pulse leads to a temporary deflection in the orientation of migratory birds. Experientia 50: 697–700.

[56] Beason RC, Dussourd N, Deutschlander ME (1995) Behavioural evidence for the use of magnetic material in magnetoreception by a migratory bird. J Exp Biol 198: 141–146. 9317510 10.1242/jeb.198.1.141

[57] Wiltschko W, Munro U, Ford H, Wiltschko R (1998) Effect of a magnetic pulse on the orientation of silvereyes, *Zosterops l. lateralis*, during spring migration. J Exp Biol 201: 3257–3261. 9808838 10.1242/jeb.201.23.3257

[58] Holland RA (2010) Differential effects of magnetic pulses on the orientation of naturally migrating birds. J R Soc Interface 7 1617–1625. 20453067 10.1098/rsif.2010.0159 PMC2988258

[59] Munro U, Munro JA, Phillips JB, Wiltschko R, Wiltschko W (1997) Evidence for a magnetite-based navigational “map” in birds. Naturwissenschaften 84: 26–28.

[60] Munro U, Munro JA, Phillips JB, Wiltschko W (1997) Effect of wavelength of light and pulse magnetisation on different magnetoreception systems in a migratory bird. Aust J Zool 45: 189–198.

[61] Mouritsen H, Mouritsen O (2000) A mathematical expectation model for bird navigation based on the clock-and-compass strategy. J Theor Biol 207: 283–291. 11034834 10.1006/jtbi.2000.2171

[62] Holland RA, Thorup K, Gagliardo A, Bisson IA, Knecht E, et al (2009) Testing the role of sensory systems in the migratory heading of a songbird. J Exp Biol 212: 4065–4071. 19946085 10.1242/jeb.034504

[63] Gagliardo A, Ioalè P, Savini M, Wild JM (2006) Having the nerve to home: trigeminal magnetoreceptor versus olfactory mediation of homing in pigeons. J Exp Biol 209: 2888–2892. 16857872 10.1242/jeb.02313

[64] Gagliardo A, Ioalè P, Savini M, Wild M (2008) Navigational abilities of homing pigeons deprived of olfactory or trigeminally mediated magnetic information when young. J Exp Biol 211: 2046–2051. 18552292 10.1242/jeb.017608

[65] Gagliardo A, Ioalè P, Savini M, Wild M (2009) Navigational abilities of adult and experienced homing pigeons deprived of olfactory or trigeminally mediated magnetic information. J Exp Biol 212: 3119–3124. 19749104 10.1242/jeb.031864

[66] Wiltschko R, Schiffner I, Fuhrmann P, Wiltschko W (2010) The role of magnetite-based receptors in the beak in pigeon homing. Curr Biol 20: 1534–1538. 20691593 10.1016/j.cub.2010.06.073

[67] Wallraff HG (1999) The magnetic map of homing pigeons: an evergreen phantom. J Theor Biol 197: 265–269. 10074399 10.1006/jtbi.1998.0874

[68] Patzke N, Manns M, Güntürkün O, Ioalè P, Gagliardo A (2010) Navigation-induced ZENK expression in the olfactory system of pigeons (*Columba livia*). Eur J Neurosci 31: 2062–2072. 20529114 10.1111/j.1460-9568.2010.07240.x

[69] Rastogi A, Kumari Y, Rani S, Kumar V (2011) Phase inversion of neural activity in the olfactory and visual systems of a night-migratory bird during migration. Eur J Neurosci 34: 99–109. 21676040 10.1111/j.1460-9568.2011.07737.x

[70] Biro D, Guilford T, Dell’Omo G, Lipp H-P (2002) How the viewing of familiar landscapes prior to release allows pigeons to home faster: evidence from GPS tracking. J Exp Biol 205: 3833–3844. 12432007 10.1242/jeb.205.24.3833

[71] Guilford T, Åkesson S, Gagliardo A, Holland RA, Mouritsen H, et al (2011) Migratory navigation in birds: new opportunities in an era of fast-developing tracking technology. J Exp Biol 214: 3705–3712. 22031734 10.1242/jeb.051292

[72] Wallraff H (2005) Avian navigation: pigeon homing as a paradigm. Berlin: Springer Verlag. 237 p.

[73] Walcott C (1978) Anomalies in the Earth’s magnetic field increase the scatter of pigeons’ vanishing bearings. In: Schmidt-Koenig K, Keeton WT, editors. Animal migration, navigation and homing. New York: Springer. 143–151.

[74] Walcott C (1980) Effects of magnetic fields on pigeon orientation. In: Nohring R, editor. Acta XVII Congressus Internationalis Ornithologici I. Deutsche Ornithologen-Gesellschaft. 588–592.

[75] Fransson T, Jakobsson S, Johansson P, Kullberg C, Lind J, et al (2001) Magnetic cues trigger extensive refuelling. Nature 414: 35–36. 11689932 10.1038/35102115

[76] Henshaw I, Fransson T, Jakobsson S, Kullberg C (2010) Geomagnetic field affects spring migratory direction in a long distance migrant. Behav Ecol Sociobiol 64: 1317–1323.

[77] Åkesson S, Alerstam T (1998) Oceanic navigation: are there any feasible geomagnetic bi-coordinate combinations for albatrosses? J Avian Biol 29: 618–625.

[78] Boström JE, Åkesson S, Alerstam T (2012) Where on earth can animals use a geomagnetic bi-coordinate map for navigation? Ecography 35: 1039–1047.

[79] Muheim R, Phillips JB, Åkesson S (2006) Polarized light cues underlie compass calibration in migratory songbirds. Science 313: 837–839. 16902138 10.1126/science.1129709

[80] Dennis TE, Rayner MJ, Walker MM (2007) Evidence that pigeons orient to geomagnetic intensity during homing. Proc R Soc Lond B 274: 1153–1158. 10.1098/rspb.2007.3768 PMC2189574 17301015

[81] Åkesson S, Morin J, Muheim R, Ottosson U (2001) Avian orientation at steep angles of inclination: experiments with migratory white-crowned sparrows at the magnetic North pole. Proc R Soc B 268: 1907–1913. 10.1098/rspb.2001.1736 PMC1088826 11564346

[82] Kaiser A (1993) A new multi-category classification of subcutaneous fat deposits of songbirds. J Field Ornithol 64: 246–255.

[83] Chernetsov N (1999) Timing of spring migration, body condition, and fat score in local and passage populations of the Reed Warbler *Acrocephalus scirpaceus* on the Courish Spit. Avian Ecol Behav 2: 75–88.

[84] Chernetsov N, Kishkinev D, Kosarev V, Bolshakov CV (2011) Not all songbirds calibrate their magnetic compass from twilight cues: a telemetry study. J Exp Biol 214: 2540–2543. 21753048 10.1242/jeb.057729

[85] Mouritsen H, Larsen ON (1998) Evaluating alternative methods for the analysis of Emlen funnel data. In: Mouritsen H, editor. Compasses and orientational strategies of night migrating passerine birds. Odense: Center of Sound Communication, Institute of Biology, Odense University, PhD Thesis. 115–124.

